# Bio-Imaging of Colorectal Cancer Models Using Near Infrared Labeled Epidermal Growth Factor

**DOI:** 10.1371/journal.pone.0048803

**Published:** 2012-11-08

**Authors:** Gadi Cohen, Shimon Lecht, Hadar Arien-Zakay, Keren Ettinger, Orit Amsalem, Mor Oron-Herman, Eylon Yavin, Diana Prus, Simon Benita, Aviram Nissan, Philip Lazarovici

**Affiliations:** 1 School of Pharmacy Institute for Drug Research, Faculty of Medicine, The Hebrew University of Jerusalem, Jerusalem, Israel; 2 Advanced Technology Center, The Chaim Sheba Medical Center, Tel-Hashomer, Israel; 3 Department of Pathology and Surgical Oncology Laboratory, Hadassah-Hebrew University Medical Center, Mount Scopus, Jerusalem, Israel; 4 Department of Surgery, Hadassah-Hebrew University Medical Center, Mount Scopus, Jerusalem, Israel; The Chinese University of Hong Kong, Hong Kong

## Abstract

Novel strategies that target the epidermal growth factor receptor (EGFR) have led to the clinical development of monoclonal antibodies, which treat metastatic colorectal cancer (mCRC) but only subgroups of patients with increased wild type KRAS and EGFR gene copy, respond to these agents. Furthermore, resistance to EGFR blockade inevitably occurred, making future therapy difficult. Novel bio-imaging (BOI) methods may assist in quantization of EGFR in mCRC tissue thus complementing the immunohistochemistry methodology, in guiding the future treatment of these patients. The aim of the present study was to explore the usefulness of near infrared-labeled EGF (EGF-NIR) for bio-imaging of CRC using *in vitro* and *in vivo* orthotopic tumor CRC models and *ex vivo* human CRC tissues. We describe the preparation and characterization of EGF-NIR and investigate binding, using BOI of a panel of CRC cell culture models resembling heterogeneity of human CRC tissues. EGF-NIR was specifically and selectively bound by EGFR expressing CRC cells, the intensity of EGF-NIR signal to background ratio (SBR) reflected EGFR levels, dose-response and time course imaging experiments provided optimal conditions for quantization of EGFR levels by BOI. EGF-NIR imaging of mice with HT-29 orthotopic CRC tumor indicated that EGF-NIR is more slowly cleared from the tumor and the highest SBR between tumor and normal adjacent tissue was achieved two days post-injection. Furthermore, images of dissected tissues demonstrated accumulation of EGF-NIR in the tumor and liver. EGF-NIR specifically and strongly labeled EGFR positive human CRC tissues while adjacent CRC tissue and EGFR negative tissues expressed weak NIR signals. This study emphasizes the use of EGF-NIR for preclinical studies. Combined with other methods, EGF-NIR could provide an additional bio-imaging specific tool in the standardization of measurements of EGFR expression in CRC tissues.

## Introduction

Colorectal cancer (CRC) is one of the most common malignancies in the Western societies. Long-term survival of CRC-diagnosed patients is correlated with disease stage at diagnosis. In early stages as well as in selected patients with advanced disease, surgery is the main modality of treatment [1]. At least 40% of patients with CRC will develop either synchronous or metachronous distant metastases, most of them will succumb to their disease and die [2]. Some of characteristics of the malignant phenotype of CRC are correlated with overexpression and hyper-activation of receptor tyrosine kinases such as epidermal growth factor receptor (EGFR), which make these receptors attractive targets for cancer treatment [3]. In most CRC patients, the progression from normal colonic mucosa to cancer involves a defined cascade of molecular changes, that spreads over years [4]. Endoscopic polypectomy was shown to reduce CRC-related mortality [5]. This procedure requires fibro-optic colonoscopy visualization of the CRC tissue followed by histological evaluation. In addition the CRC tissues are often evaluated by RT-PCR [6], immunohistochemistry [7] and *in situ* hybridization [8] techniques, which showed a much higher degree of discordance between primaries and related CRC metastases [9].

EGFR is frequently overexpressed in a variety of solid tumor, of the brain, breast, lung, ovary and pancreas, and is associated with increased metastatic potential and poor prognosis of CRC [10]. Biological agents that inhibit EGFR have demonstrated clinical activity as single agents or in combination with chemotherapy, the most promising of these agents being cetuximab and panitumumab. Unfortunately, these antibodies are clinically effective in only a minority of patients with CRC [11]. The clinical success of these monoclonal antibody therapies is uniformly limited by the development of acquired resistance to EGFR blockade [12]. One mechanism to resistance was recently elucidated: cetuximab resistant cells contain an EGFR mutation in the extracellular domain (S492R) that impairs cetuximab, but not epidermal growth factor (EGF) binding [12]. Therefore, since the response to therapy require the EGFR target to be present, the development of BOI methods for quantitative detection of EGFR protein levels in CRC primary and secondary tumor tissues is necessary, in order to guide the treatment of individual selected for EGFR targeted antibody treatment and in particular those who relapse while on EGFR targeting therapies [13]. The advent of EGFR-targeted antibodies, cetuximab and panitumumab has paved the way to individualized medicine of mCRC. Current data suggests that the evaluation of KRAS and bRAF mutation and PI3K/PTEN alteration could be useful for selecting patients who are unlikely to respond to anti-EGFR-targeted antibodies. It was found that responsive CRC tumors carry wild type KRAS/bRAF and tend to have a modest, increase copy number of the EGFR gene, which is translated into a modest increase in EGFR level. Therefore, the EGFR gene copy number detection and quantitative evaluation of EGFR protein level will likely improve tailoring of cetuximab and panitumumab therapies for mCRC patients [12]. However, the technical difficulties of the immunohistochemistry technique, which is used to assess the expression of the EGFR in fixed tissues, may have limited the detection of small EGFR protein level increase so far [11]. Therefore, novel sensitive BOI methods of EGFR protein level in mCRC are needed. EGFR scintigraphy, represents such a method which is based on the binding, internalization, and retention of the radiolabeled EGFR-targeted agents in intracellular compartments and has been demonstrated with radiolabeled EGF and with radiolabeled monoclonal antibody directed against EGFR [14]. However, the disadvantage of these methods is the use of radioactive materials.

Near infrared (NIR) optical BOI offers unique advantages for diagnostics of mCRC: it offers high sensitivity, it can be used with different NIR tags and it can provide dynamic, real time *in vitro* and *in vivo* images by non radioactive means [15]. NIR light (700–1000 nm wavelength) can penetrate into tissue, and offers a potentially safe, noninvasive method of characterizing tumors [16]. In most applications, NIR BOI is used for assisting targeted fluorescent contrast agents that not only provide enhanced contrast, but also, more importantly, reveal specific molecular events associated with CRC tumor initiation and progression [17]. Recent studies have established the use of Affibody-mediated targeting of NIR excitable fluorescent contrast agents for the detection of malignant cells and tumors [18].

Therefore, the aims of the present study were to develop and characterize novel *in vitro* CRC models that resembled CRC heterogeneity and to assess whether it is possible to quantify the level of EGFR in *ex vivo* fresh CRC tissue samples, orthotopic tumor in mice and newly developed cell lines models by using EGF conjugated with IRDye 800CW (EGF-NIR) probe. We found optimal conditions for BOI of EGFR using EGF-NIR probe in these models, applicable for endoscopic and Odyssey Infrared Imager analyses. Furthermore, by using image processing analysis and western blotting we confirmed that the intensity of EGF-NIR signal to background ratio reflects EGFR protein level in the *in vitro* CRC models and *in situ* human CRC tissues investigated.

**Figure 1:**
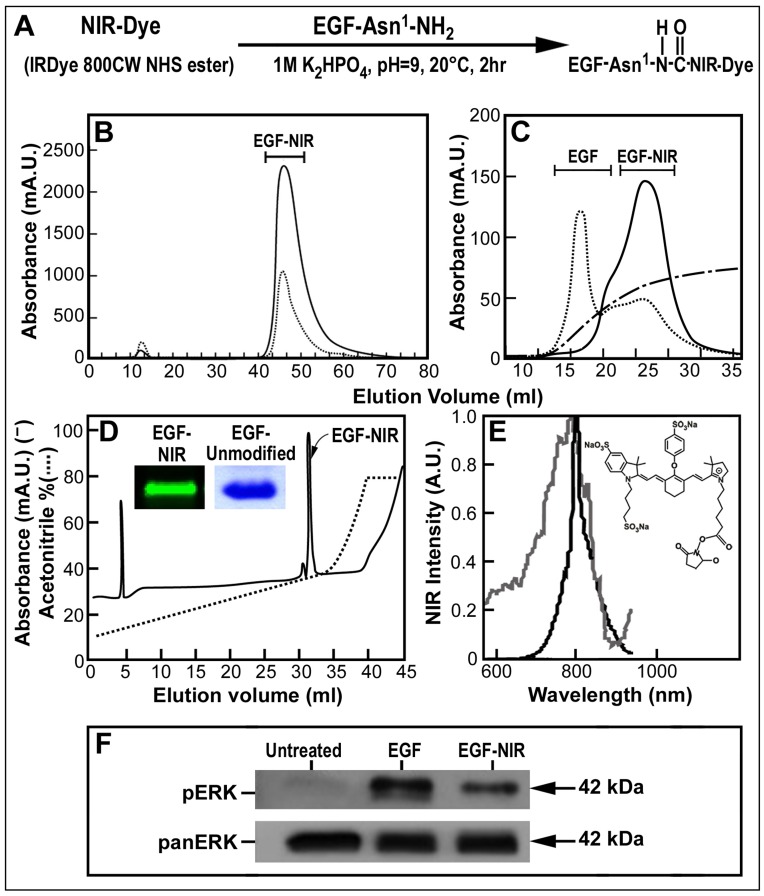
Synthesis, purification, spectrum, electrophoresis properties and signaling of EGF-NIR. (A) Reaction scheme for the synthesis of EGF-NIR conjugate, the first amino acid asparagine at the amino terminal is indicated as Asn1. (B) Separation of synthesis reaction mixture on gel permeation chromatography and of EGF-NIR sample from gel permeation on (C) anion exchange chromatography; EGF-NIR-full line (800 nm); gradient of NaCl-broken line; unconjugated EGF-dotted line. (D) HPLC separation of EGF-NIR purified from anion exchanger chromatography. Full line represents absorbance at 226 nm and dotted line indicates the gradient. Insert-12% SDS-PAGE analysis of 10 µg of EGF-NIR scanned with Odyssey and unmodified EGF stained with coomassie blue. (E) NIR spectrum of EGF-NIR [excitation (gray line) and emission (black line)]; Insert-IRDye 800CW NHS ester; (F) EGF-NIR induced Erk phosphorylation.

**Figure 2:**
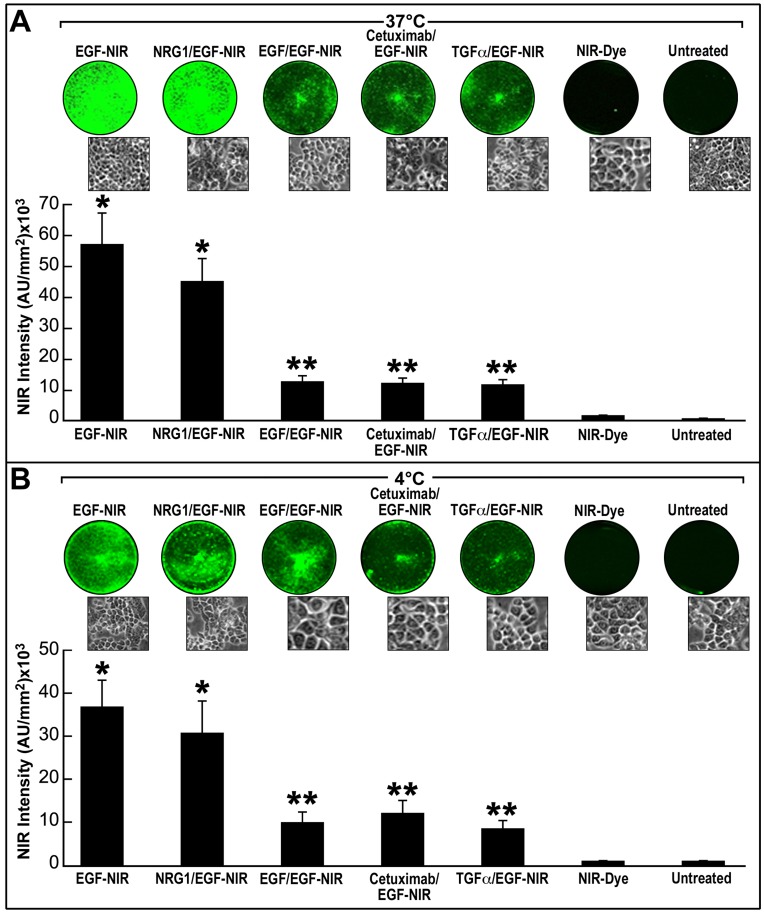
The specificity and selectivity of EGF-NIR probe binding measured by IC-NIR imaging. HT-29 cells were incubated for 15 minutes at 37°C (A) and 4°C (B) with 7 nM EGF-NIR in the presence or absence of 100 nM EGF. Competition experiments with 500 nM of cetuximab, TGF-α or NRG1 were also conducted. In control experiments the cultures were incubated with 7 nM NIR-Dye to evaluate nonspecific labeling of the cells. The NIR intensity at 800 nm was estimated under identical conditions for all cultures and the mean ± SD (n = 9) is presented. Upper inserts: NIR scans; lower inserts: phase-contrast photomicrographs of the cultures,* p<0.05 vs. NIR-Dye; ** p<0.05 vs. EGF-NIR.

**Figure 3:**
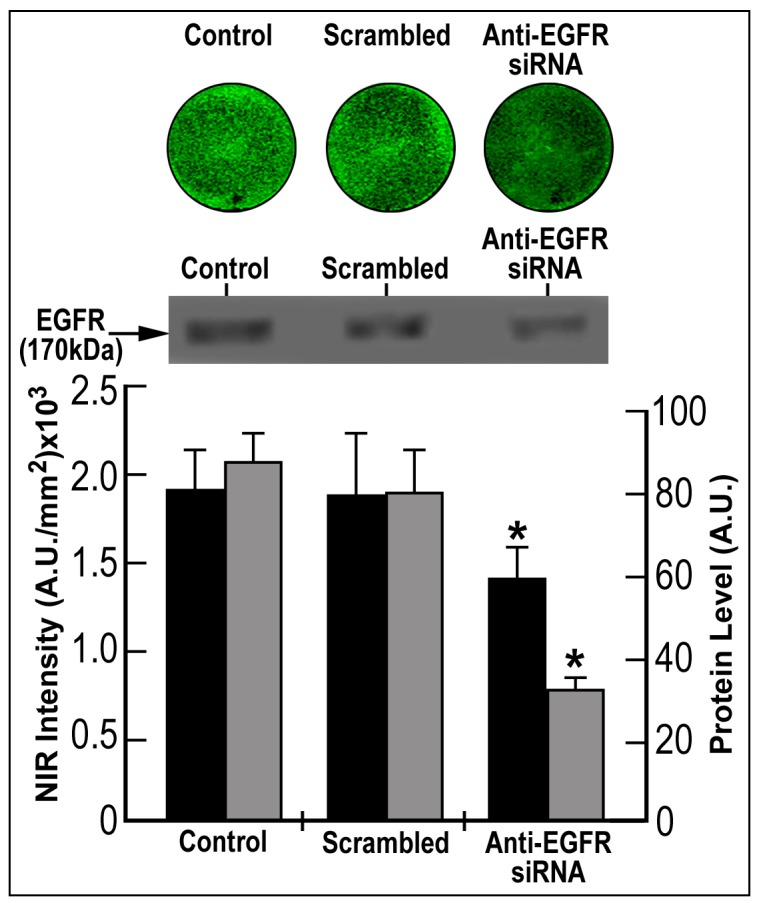
siRNA-induced knock down of EGFR evaluated by IC-NIR imaging. HT-29 cells were transfected for 2 days with 5 nM anti-EGFR Silencer select siRNA or scrambled RNA or left untreated (control). Evaluation of EGFR expression was performed by In Cell NIR imaging using 7 nM EGF-NIR (black bars) and western blotting (gray bars). The values are mean ± SD (n = 3). * p<0.05 vs. scrambled or control.

## Materials and Methods

### Cell Culture

Human colon carcinoma cell lines HT-29, SW620, COLO205, A431 human epithelial squamous carcinoma cells and rat small intestine epithelial cell clone IEC 6 were purchased from American Type Culture Collection (ATCC, Manassas, VA) and adjusted for growth in Dulbecco’s Modified Eagle’s Medium (DMEM) containing 10% fetal bovine serum, 2 mM L-glutamine and 10000 U/ml penicillin and 100 µg/ml streptomycin. The cells were grown at 37°C, 6% CO_2_ in a humidified incubator. All experiments were carried out under GLP conditions using a clean room according to ISO7 requirements (10,000 particles/m^3^).

### Preparation, Purification and Characterization of EGF-NIR

EGF-NIR was synthesized according to LI-COR Biosciences (Lincoln, NE, USA) instructions using IRDye 800CW NHS ester (2-(3-{5-[7-(5-amino-1-carboxy-pentylcarbamoyl)-heptanoylamino]-1-carboxy-pentyl}ureido)-pentanedioic acid) for conjugation to human recombinant EGF (Peprotech, Asia, Rehovot, Israel). Briefly, EGF (32 nmole) was incubated with 5 equivalents of IRDye 800CW NHS ester in 1 M K2HPO4, pH 9.0, for 2 hours in darkness at 20°C, with stirring. Following conjugation, the coupling mixture of free reagents and EGF-NIR was applied to Hiprep 26/10 (Fine Sephadex G-25, particles size 90 µm) desalting column (GE Healthcare, Life sciences, Buckinghamshire, UK) of a volume of 55 ml (Vo = 15 ml). This column was equilibrated and eluted at 10 ml/min with distilled water using FPLC AKTA P900 instrument (GE-Healthcare Life Sciences, Buckinghamshire, UK). This was followed by collecting the excluded peak and adjusting it to pH 8.0 by addition of 1 ml of 0.02 M Tris HCl buffer (pH 8.0). The solution was applied for anion exchange chromatography on Hitrap DEAE FF (DEAE Sepharose High Flow, GE Healthcare, Life sciences, Buckinghamshire, UK) equilibrated with 0.02 M Tris HCl buffer (pH 8.0) at a flow rate of 1 ml/min and EGF-NIR was eluted from the column using a gradient of 1–100% NaCl in the equilibration buffer. The purified EGF-NIR was dialyzed in 3000 cut off dialysis bags (Thomas Scientific, Swedesboro, NJ, USA) for 14 hours in dark at 4°C against distilled water. The distilled solution was lyophilized, and 0.1 mg of dry samples of EGF-NIR dissolved in 1% triflouroacetic acid (TFA) were finally separated by HPLC using a Sperisorb DDS2 column (LKB instruments, Gaithersburg MD) using two linear gradients: the first gradient from 10–35%, followed by a second gradient of 35–85% acetonitrile in 1% TFA. The purification was performed at a flow rate of 4.7 ml/min (100 bar pressure). The full run continued for 45 min and the EGF-NIR peak was estimated by the optical absorbance at 226 and 700 nm. Molar concentrations of dyes and EGF were calculated using molar extinction coefficients of 270,000 M^−1^cm^−1^ for IRDye 800CW at 780 nm, and 18,000 M^−1^cm^−1^ for EGF. Absorbance at 280 nm was used to calculate EGF protein concentration based on its molar extinction coefficient. Dual wavelength absorbance was used to determine dye: protein ratio. EGF-NIR emission was measured in PBS using a Fluoro-Max 4 spectrofluorimeter (JY Horiba, Edison, NJ, USA) with a Xenon arc lamp as excitation source of 774 nm in 1 cm cuvette, and at a scanning rate of 80 nm/sec. For validation, EGF-NIR from LI-COR Biosciences (Lincoln, NE, USA) was also used. EGF-NIR was submitted for analysis on 12% SDS-PAGE by comparison with native, unlabeled EGF. Samples of 10 µg proteins were separated and visualized by commassie blue staining and the NIR emission was measured by positioning and scanning the gel in the Odyssey® Infrared Imager (LI-COR Biosciences, Lincoln, NE, USA).

### Western Blotting of EGFR and Erk Phosphorylation

The levels of EGFR in cell lines and CRC tissue and Erk phosphorylation, were estimated upon extraction with cell lysis buffer (Cell Signaling Technology, Inc. Danvers, MA, USA). The ability of EGF-NIR to stimulate ERK phosphorylation in A431 cells was compared to that of unlabeled EGF. 2×10^6^ cells at 90% confluence in a 6 well plate were serum starved for 2 hours. Starvation media was replaced with regular medium containing 7 nM EGF-NIR. Cells were incubated for 15 min at room temperature and harvested. 50 µg protein lysates were separated by 10% polyacrylamide SDS-PAGE and transferred on ice to nitrocellulose membranes (90 V for 1.5 hours; Whatman, Dassel, Germany). Non-specific binding was blocked by incubation of the membranes for 2 hours at room temperature (RT) with 5% non-fat powdered milk (Bio-Rad, Hercules, CA, USA) in Tris buffered saline containing 0.1% Tween-20. Immunodetection was performed with monoclonal anti-EGFR antibody (Cell Signaling Technology, Inc. Danvers, MA, USA) or primary antibodies (1∶1,000) against phospho-or pan-Erk1/2 (Cell Signaling Technology, Inc. Danvers, MA, USA), followed by horseradish peroxidase (Jackson ImmunoResearch, West Grove, PA, USA) and developed with ECL (Pierce, Rockford, IL, USA).

**Figure 4:**
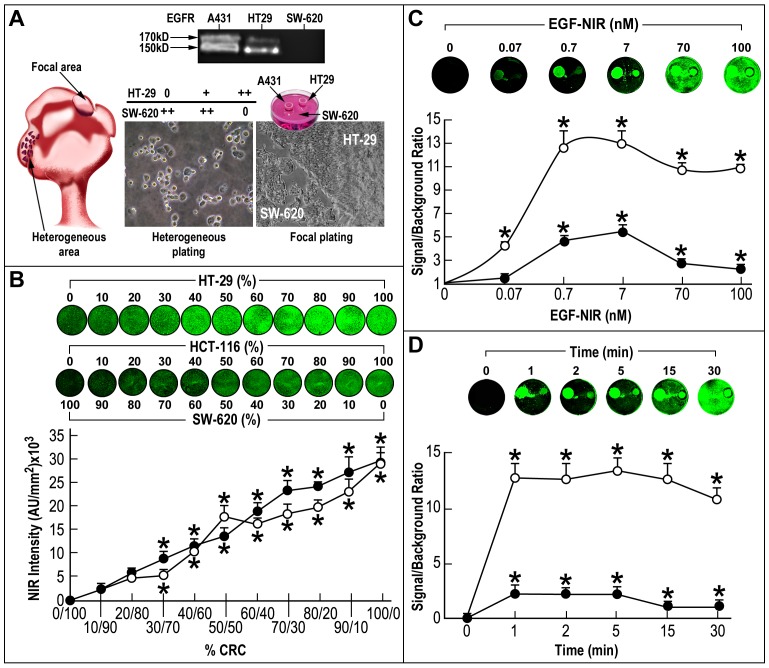
Saturation, kinetics and sensitivity of EGF-NIR binding using IC-NIR imaging of CRC cultures. (A) Left: a scheme of CRC polyp with high spots of transformed CRC cells (focal area) and heterogeneous area including both normal and transformed CRC cells; Middle: generation of a heterogeneous mixture at different ratios (%) between HT-29 and SW 620; 0 – no cells; +/++ -presence of different cell concentrations; Right: focal plating (in a ring) of HT-29 and A431 monolayer surrounded by SW620 monolayer; insert-the level of EGFR (170 kD) and non-mature EGFR (150 kD) in the cells; (B) The relationship between the NIR intensity of 15 min binding with 7 nM EGF-NIR (mean ± SD, n = 9) and the percentage of SW620 in the cell mixture with either HT-29 (closed circles) or HCT116 (open circles) * p<0.05 vs. 100% SW620; (C) The relationship between SBR (mean ± SD, n = 9) and EGF-NIR concentration; A431 (open circles); HT-29 (closed circles); binding was performed for 15 minutes. Insert: NIR scans; * p<0.05 vs. 0.01 nM; (D) The kinetics of 7 nM EGF-NIR binding (mean ± SD, n = 9) to focal cultures of A431 (open circles) or HT-29 (closed circles); Insert: NIR scans; * p<0.05 vs. 0 min.

### Preparation of *in vitro* CRC Models and in Cell NIR Imaging (IC-NIR)

#### Homogenous monolayer of an individual cell line

CRC cells were plated at a density of 150,000 cells/well in 12 wells tissue culture plates (Nunc, Rochester, NY, USA) two days before the experiments generating homogenous cell monolayer. Thereafter, the culture medium was replaced with fresh medium containing 7 nM EGF-NIR, for 15 min at 4°C and 37°C, to measure total binding. At the end of the experiment the cultures were washed three times with 1 ml PBS and cell associated NIR intensity was estimated. To evaluate the non specific binding, sister cultures were incubated with the same concentration of EGF-NIR, at the same conditions, in the presence of excess of 100 nM EGF. Specific binding by EGF-NIR imaging is defined as the difference between the NIR intensity of total binding and NIR intensity of non specific binding. Competition experiments with 500 nM of either cetuximab, transforming growth factor α (TGF-α) or neuregulin 1 (NRG1) were performed by concomitant incubation of the competitor with EGF-NIR. The results are presented as the mean ± SD of at least three independent experiments (n = 9). The NIR imaging was estimated using Odyssey Infrared Imager at the following conditions range: resolution: 170–340 microns; pixel area: 0.03 mm^2^ (approximately 15–20 cells); quality: medium-low; focus offset: 1–3; channels: 800 nm; intensity: 1–3.

#### Heterogeneous focal monolayer of CRC cells

To mimic high spots of transformed CRC cells in the polyp [19] and to enable direct measurements of signal to background ratio in the same experiment, a focal cell culture approach was used. Different colorectal cancer cell lines (with different levels of EGFR) or 15×10^3^ A431 (high levels of EGFR) were plated to confluence inside a 4 mm inner diameter cloning ring (Sigma-Aldrich, St Louis, MO, USA) placed at the center of a well. Cells were left to adhere for 2 hours in an incubator. Thereafter, 15×10^3^ SW620 cells (lacking EGFR) were plated in the cell-free area surrounding the cloning ring and left to adhere for 2 hours at the same conditions. Two types of such experiments were performed: a. one focus. b. two foci. At the end of cell adherence step, the cloning ring was removed and the cells were washed with culture medium. Two days after generating the model, the cultures were subjected to binding and imaging experiments as previously described. Signal/Background ratio (SBR) values indicate the ratio of NIR fluorescent signal of the central circle (focal area of CRC or A431 cells) to NIR fluorescent signal of outside area (SW620).

**Figure 5:**
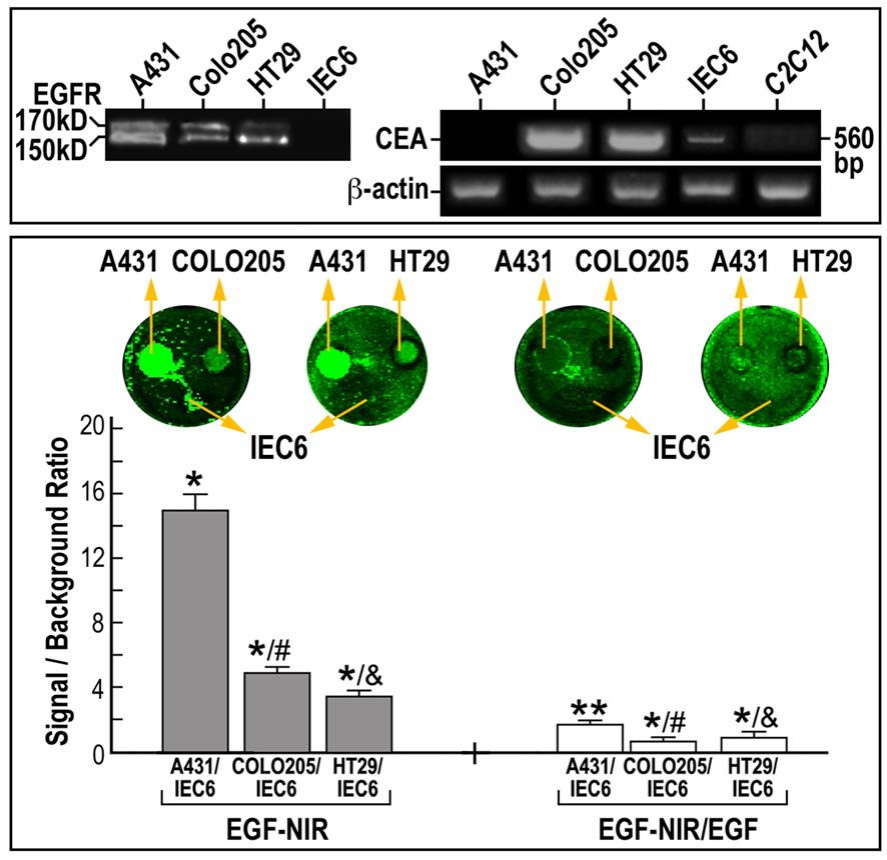
IC-NIR BOI of EGFR levels in CRC clones, in relation to the level of expression of CEA. Different CRC cells were focally plated on a background of IEC6 enterocyte monolayer. The cultures were incubated for 15 min at 37°C with 7 nM EGF-NIR in the presence (nonspecific binding – white bars) or absence (total binding – grey bars) of 100 nM unmodified EGF. The signal (CRC cell line)/ background (IEC6) ratio was estimated at identical conditions for all cultures and is presented as the mean ± SD (n = 9). Significance: * p<0.05 compared to IEC6 values; ** p<0.05 compared to total binding of the respective group p< 0.05 compared to A431 and & p<0.05 compared to COLO 205; Lower inserts: NIR scans; Upper inserts: left-EGFR protein expression by western blotting; arrow indicate the position of mature EGFR 170 kD protein and non-glycosylated 150 kD protein; right-mRNA expression of CEA and β-actin in cell cultures.

#### Heterogeneous suspensions of CRC cells

To mimic heterogeneous distribution of transformed CRC cells in the polyp [20] and to enable evaluation of sensitivity of detection of the minimal amount of EGFR expressing cells among control normal enterocytes, heterogeneously mixed cultures of HT-29 and SW620 cells were prepared. Suspension cultures of HT-29 were mixed with suspension cultures of SW620 to generate different percentage of the individual cell culture in the same volume. SW620 cells could be distinguished from HT-29 cells by their elongated morphology. At conditions designated as “0%” the suspension contains 0% HT29 cells and 100% SW620 cells, and at “100%”, the suspension contains 100% HT-29 cells and 0% SW620 cells. In the other cases the percentages indicate the ratio between percentage HT-29 cells and percentage of SW620 cells. The binding experimental conditions and measurements of NIR fluorescent intensity (arbitrary units/mm^2^) of the different heterogeneous cultures was performed as above.

**Figure 6:**
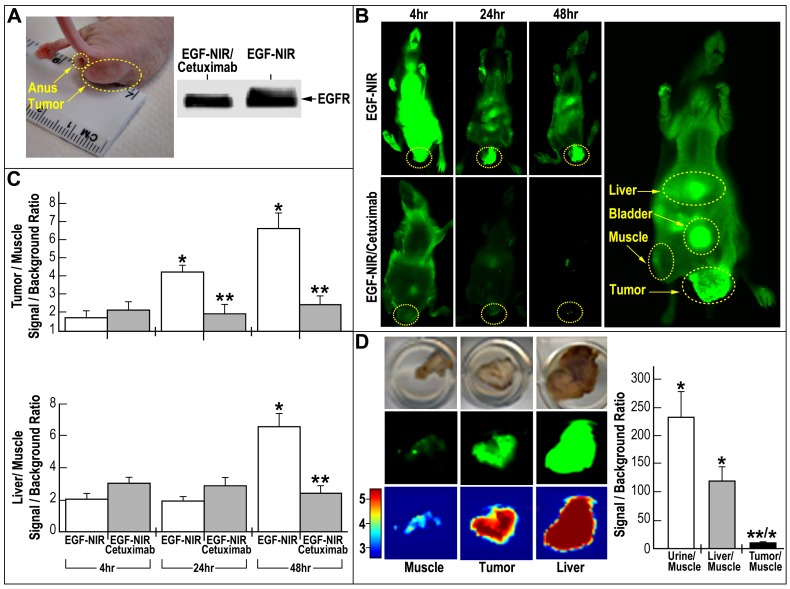
Whole body *in vivo* and isolated tissue *ex vivo* EGF-NIR BOI of mice with HT-29 orthotopic tumors. (A) Photograph of an orthotopic tumor and EGFR protein expression in the tumors; (B) Time course of EGF-NIR accumulation in tissues of tumor-bearing mice. The mice were injected i.v. with 1 nmol of EGF-NIR in untreated mice (upper row, n = 6) or mice pre-injected with 1 µg/ml cetuximab (lower row, n = 4); high resolution BOI of a mouse injected with EGF-NIR and circles indicate ROI measurements (C) Time course of tissue accumulation of EGF-NIR at 48 hours from mice presented in B; Signal intensity at 800 nm were normalized to background fluorescence using an arbitrary tumor circle (10–20 ROIs/mouse) compared to an identical area on the flank (adjacent muscle); * p<0.05 compared to EGF-NIR 4 hours; ** p<0.05 compared to mice injected with EGF-NIR; (D) EGF-NIR signal/background ratio in isolated tissue from the tumor-bearing mice 48 hours after injection. * p<0.05 compared to muscle, ** p<0.05 compared to liver; Insert: Upper-photographs of tissues in the dish; Middle-NIR images; Lower-spectral intensity maps; Intensity scale-red-brown (5) high expression; blue (3) very low expression.

### RT-PCR and EGFR siRNA Silencing

Total RNA was isolated and genomic DNA was degraded from the RNA preparations, using the SV total RNA isolation system (Qiagen GmbH, Hilden, Germany). 1 µg of total RNA was reverse transcribed (Promega, Madison, WI, USA), according to the manufacturer’s instructions. PCR was performed in a final volume of 50 µl containing 5 µg cDNA, 50 pmol of each upstream sense and downstream sense primers of CEA or EGFR [21], [22], and 25 µl of GoTaq® Green Master Mix (Promega, Madison, WI). PCR experiments were conducted for 35 cycles. To generate various cDNA fragments, a Mastercycler gradient (Eppendorf, Hamburg, Germany) was programmed as follows: denaturation at 95°C for 1 min, annealing at 61°C and elongation at 72°C for 1 min. To knock down EGFR the standard amine transfection agent protocol of Ambion (Applied Biosystem, Austin, TX, USA) was followed. Briefly, 5 nM of 21 mer anti-EGFR Silencer select small interference RNA (siRNA) and scrambled RNA were reverse transfected into A431 cell cultures using siPORTNeoFX transfection agent according to manufacturer protocol. The cells at a density of 80,000 cells/ml were applied on 12 well plates and 2 days after transfections were analyzed. Knock down of EGFR mRNA was confirmed by Western blotting. The following carcino embryonic antigen (CEA) and EGFR primers, prepared by SyntezzaBioScience Ltd., Jerusalem, Israel, were used:

- Human CEA CAM5: Sense: 5′-CGCATACAGTGGTCGAGAGA-3′; Antisense: 5′-ATTGCTGGAAAGTCCCATTG-3′
- Rat CEA1: Sense: 5′-CTACAGGCTGAGGGATGCTC-3′ Antisense: 5′-GGTCCCGTCACAGTTACGTT-3′
- Human EGFR: Sense: 5′-CGAGGGCAAATACAGCTT-3′ Antisense: AAATTCACCAATACCTATT-3′
- Human EGFR siRNA: Sense: 5′-CCAUAAAUGCUACGAAUAUtt-3′ Antisense: 5′-AUAUUCGUAGCAUUUAUGGag-3′
- Scrambled siRNA: Sense: 5′-UAACGACGCGACGACGUAATT-3′ Antisense: 5′-UUACGUCGUCGCGUCGUUATT-3′

### Preparation and NIR Imaging of CRC Orthotopic Tumors in Mice

This study using Male Balb/c nude (Harlan, Israel) mice was approved, performed and supervised by the guidelines of The Chaim Sheba Medical Center Animal Care and Use Committee. HT-29 cells were trypsinized, washed and resuspended at concentration of 1×10^7^ cells/ml in PBS. For tumor implantation, the mice were anesthetized by intra-peritoneal injection of a mixture of ketamine (100 mg/kg) and xylazine (20 mg/kg). Trans-anal injection of 1×10^6^ HT-29 cells was performed under microscope magnification (X40) using a 27 g needle. The injection was directed submucosally into the distal, posterior rectum, approximately 2–3 mm beyond the anal canal and into the rectal mucosa [23]. Mice were monitored two times weekly for tumor initiation and progression. Tumors reached ∼ 0.75 cm in size at 3–4 weeks. *In vivo* imaging of EGF-NIR fluorescence in mice was performed with a LI-COR Biosciences small-animal imager Odyssey MousePOD**®**. To visualize the tumors, 1 nmol of EGF-NIR in 100 µl saline was injected via the tail vein into tumor-positive mice in the presence (n = 4) or absence (n = 6) of cetuximab (1 µg/ml) and evaluated for systemic clearance by NIR imaging at intervals of 1–8 hours over a period of three days, after which time >95% of the signal had cleared. The mice were imaged up to two days post injection and euthanized. Statistical analysis of the images for each mouse was normalized using the same intensity scales, under the conditions previously described. SBR was calculated as follow: mean NIR intensity of the tumor divided by mean NIR intensity of the background of the adjacent muscle. Regions of interest (ROI) with identical areas were used for both tumor and background. The standard deviation of mean backgrounds was calculated using 10–20 ROIs. Due to tumor size differences between the animals receiving EGF-NIR, tumor signal divided by the background signal of similar size ROI corrected for area (pixels), provided the tumor two dimensional (2D) total labeling. Tumors, skeletal muscle and liver tissue of euthanized mice were dissected and their urine samples were collected. The tissues were weighted and introduced into plastic tubes dishes. The dishes were scanned on Odyssey Infrared Imager. NIR intensity of ROI of the tumor was compared to adjacent muscle to generate SBR. Isolated tissue analyses were performed by scanning at 800 nm channel, for the tissue accumulated EGF-NIR fluorescence signal and the SBR was calculated. The NIR imaging was estimated at the following conditions: resolution: 170–340 microns; pixel area: 0.03 mm^2^; quality: medium-low; focus offset: 1–3; channels: 800 nm; intensity: 1–3.

### Patients and CRC Tissue Specimen Collection

18 patients over the age of 18 years with histologically confirmed primary adenocarcinoma of the colon were offered participation in the study. 5 patients who received prior radiation or chemotherapy were ineligible for the study. The study protocol was approved by the Institutional Review Board (IRB, Helsinki Committee) of Hadassah-Hebrew University Medical Center. All samples were obtained from consenting study subjects undergoing surgical tumor resection who signed a written informed consent. All specimens underwent routine macroscopic and microscopic analysis by a board certified pathologist according to the College of American Pathologists (CAP) guidelines of histopathology reporting (www.cap.org). Tissues identified by the study pathologist as colonic adenocarcinoma and adjacent tissues [24] were then used for IC-NIR imaging.

**Figure 7:**
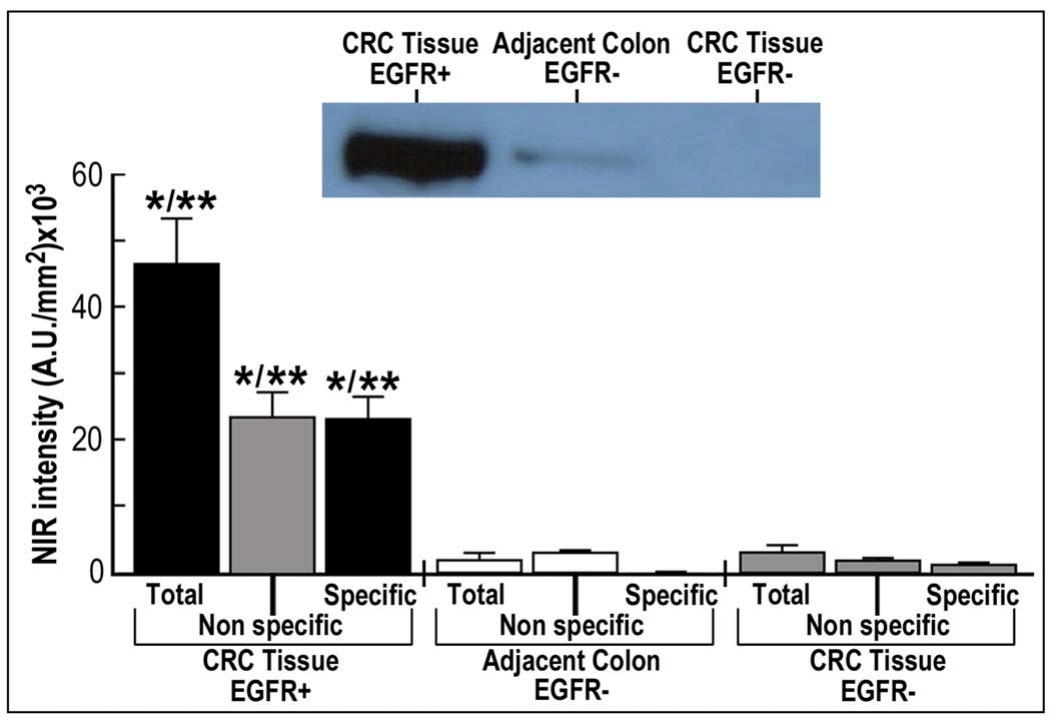
BOI of EGF-NIR binding in human CRC tissues. 36 Slices of CRC tissues and 19 slices of adjacent colon tissue (n = 10–15 ROI in each slice) were submitted for *ex vivo* binding assay for 45 min at 37°C with 70 nM EGF-NIR in the presence (non specific) or absence (total binding) of 1 µM unlabeled EGF. The NIR intensity was estimated at identical conditions for all slices (n = 12). Significance: * p<0.01 compared to respective group in adjacent colon EGFR-, ** p<0.05 compared to respective group in CRC tissue EGFR-; Insert: typical western blotting for EGFR of the slices investigated.

**Figure 8:**
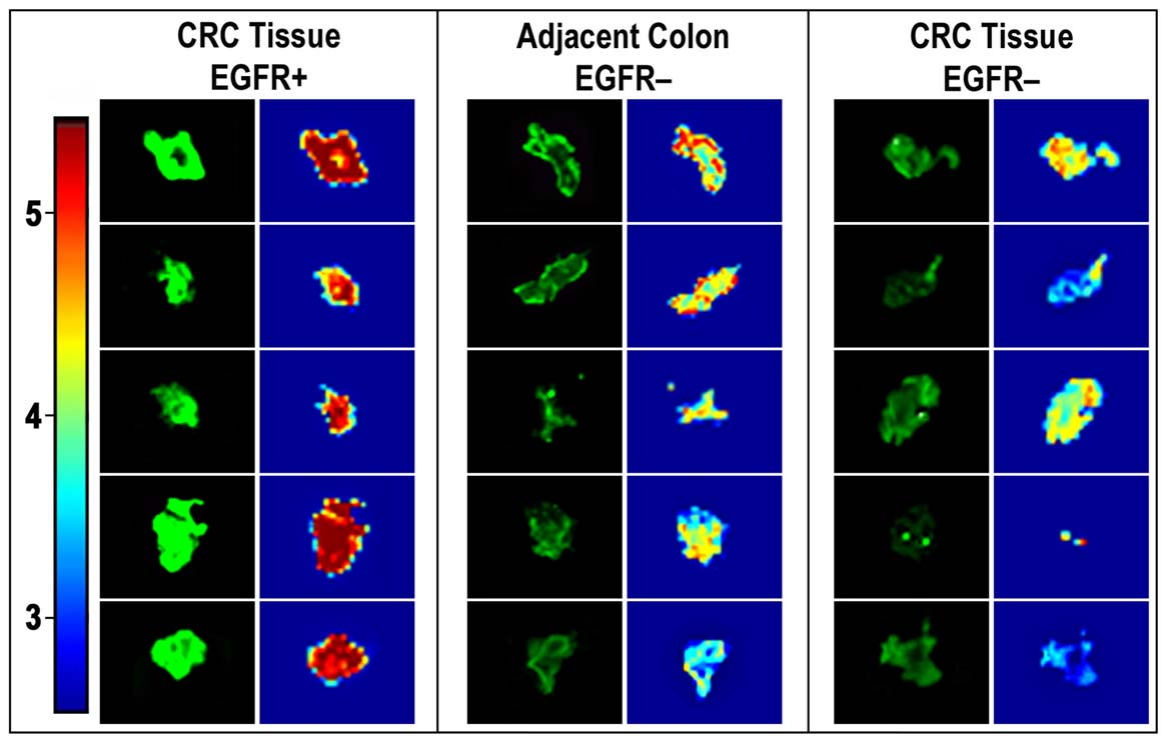
Spectral intensity maps of BOI images of specific EGF-NIR binding in human CRC tissues. Images of five typical slices, specifically labeled with EGF-NIR, as described in legend for Fig. 7. The 800 nm Odyssey Infrared Imager acquired images were processed using applied spectral imaging software, Spectral View. Intensity scale-red-brown (5) high expression of EGF-NIR binding; green (4) intermediate expression; blue (3) very low expression.

### NIR BOI of CRC Tissues

Fresh tumor tissue or adjacent colon tissues were divided in horizontal slices, 230 µm thick, which were prepared using a vibratome VT1000S (Leica, Nussloch, Germany) and incubated in ice-cold DMEM binding solution. The slices were transferred to 24 well tissue culture plates filled with DMEM saturated with 95% O_2_ and 5% CO_2_ similar to conditions enabling rectal organ cultures [25]. The plates were maintained on ice for 45 min duration in DMEM and the binding experiment was performed by addition of 70 nM EGF-NIR in the presence (non specific binding) or absence (total binding) of 1 µM of unmodified EGF. From each tissue, triplicate slices were incubated with 70 nM NIR-Dye (IRDye800CW) to evaluate the non specific binding of the dye. The binding experiment was terminated by washing the tissue three times with cold PBS. The wet slices were transferred to new 24-well plates in 1 ml/well PBS and scanned for NIR imaging intensity using Odyssey Infrared Imager, under the conditions described above. Serially, over a two years period, 43 CRC and 23 adjacent colon tissue samples were evaluated. ROIs with identical areas were used for slices from the different experimental groups. The means and standard deviations of NIR intensity (arbitrary fluorescent units/mm^2^ area) were calculated using 10–15 ROIs in each individual slice. Each slice submitted for the EGF-NIR binding experiment was also evaluated after the imaging for EGFR expression by western blotting. The data achieved was categorized according to EGFR positive CRC tissues, EGFR negative CRC tissues and adjacent colon tissue which in a majority were EGFR negative. The lack of EGFR was proved by Western blotting. 85% of the slices were included in the statistical analyses according to the following pharmacological criteria: a. total binding; b. specific binding were higher than the value of non specific absorption of NIR-Dye. The slices images were processed by high resolution imaging [26] using applied spectral imaging software, Spectral View^TM^ (ASI, Migdal Ha’Emek, Israel).

### Statistical Analysis

All results are presented as the mean ± SD of large series of independent experiments using different batches of EGF, cells, CRC and mice tumor tissues. All data were evaluated using the InStat statistics program (GraphPad, La Jolla, CA, USA). Statistically significant differences between experimental groups were determined by analysis of variance (ANOVA) with Bonferroni post-hoc test and considered significant when p<0.05.

## Results

### Preparation, Characterization and Validation of EGF-NIR Probe

The reaction scheme for synthesis of EGF-NIR is shown in Figure 1A: EGF molecule has one free amino group available for conjugation which is located at the amino-terminal of the protein and can be conjugated with IRDye800CW [27], [28]. After reaction with these reagents the EGF-NIR was purified on size exclusion (Fig. 1B) followed by anion exchange chromatography (Fig. 1C). Final purification of the EGF conjugate was achieved by high pressure liquid chromatography (Fig. 1D) resulting with a single peak found very homogenous upon SDS-PAGE electrophoresis and NIR visualization (Fig. 1D-insert). EGF absorbance and excitation spectra clearly indicates the presence of IRDye800CW (Fig. 1E) in the conjugate. Compared to the unmodified EGF, EGF-NIR stimulated EGFR resulting with increased phosphorylation of Erk (Fig. 1F). These findings indicate that the modified EGF preserved bioactivity, as evident from the ability to stimulate EGFR-induced Erk signaling pathway. Validation of EGF-NIR binding properties on CRC cell line was performed using HT-29 cultures expressing relative high level of EGFR. Comparison of NIR intensity signals of homogeneous monolayer cultures of HT-29 incubated with EGF-NIR in the presence or absence of different ligands at 4°C (Fig. 2B) and 37°C (Fig. 2A) conditions, reflects EGF-NIR cell surface binding and ligand-induced receptor internalization, respectively. Furthermore, the results clearly indicate specific and selective binding of EGF-NIR probe, similar to unmodified EGF, as concluded from lack of competition with NRG1 and partial competition with EGF, TGF-α and cetuximab.To demonstrate a direct relationship between EGFR level and binding of the EGF-NIR, we used siRNA anti-EGFR to knockdown the receptor. The efficiency of siRNA was validated using RT-PCR and indicated a knock down of ∼ 50% of EGFR mRNA. EGFR protein level, as evident from the western blotting and EGF-NIR binding, was reduced by 65% and 35% (p < 0.05), respectively, as compared to untreated controls or cultures transfected with scrambled RNA (Fig. 3), as previously documented with other cells [22]. Therefore, we conclude that the decrease NIR intensity (decreased specific binding of EGF-NIR) reflects the reduced expression of EGFR protein level.

### Development of Novel CRC *in vitro* Models for BOI

To mimic high spots of transformed CRC cells in the tumor, a focal cell culture approach (Fig. 4A-focal plating) was developed using HT-29 cells expressing high level of EGFR and SW620 lacking EGFR (Fig. 4A-insert) and for comparative purposes A431 overexpressing EGFR. In another approach to mimic clinical presentation of diffused tumor cells, a suspension of both types of above cells, at different ratios was prepared and plated (Fig. 4A-heterogeneous plating). Heterogeneous mixed cultures of HT-29 or HCT116 and SW620, at different ratios were incubated with 7 nM EGF-NIR for 15 min (Fig. 4B). It is evident that the presence of 15–30% HT-29 or HCT116 cells in a heterogeneous mixture with SW620 cells, represented the threshold of detection of the minimal amount of EGFR expressing cells among EGFR negative cells, providing a significant NIR intensity signal (Fig. 4B). Using the focal culture model, saturation (Fig. 4C) and time-course (Fig. 4D) binding experiments were preformed using IC-NIR imaging of the CRC HT-29 cell line (EGFR++) compared to A431. As found for A431, 7 nM EGF-NIR induced maximal SBR, however the maximal binding was optimal between 1 and 5 min and thereafter decreased due to increased nonspecific binding to SW620. The lower SBR in CRC HT29 compared to A431 in the different experiments is in direct correlation with the lower level of EGFR in the cells. Based on these experiments, the optimal conditions for IC-NIR imaging with the focal and heterogeneous cultures models using either A431 or HT-29 cells were setup on 7 nM EGF-NIR and 15 min of incubation since at higher concentrations of EGF-NIR or time of incubation the SBR decreased due to the increased background (increased nonspecific binding of EGF-NIR).

Since EGFR-targeting therapies are currently in use for the treatment of metastatic CRC [29] the above in vitro CRC models may be considered for BOI. To further evaluate the relationship between the EGFR level and SBR using EGF-NIR we took advantage of a panel of human CRC cell lines with different expression levels of EGFR and CEA (Fig. 5, top). Using the focal model, we performed IC-NIR BOI with these cell lines which also exhibit highly variable growth and metastatic capacities [30]. For comparison purposes COLO 205 (EGFR++) and A431 (EGFR+++) or HT 29 cells (EGFR++) were plated in different rings surrounded by small intestine epithelial IEC6 cells (EGFR -). The binding experiment was performed in the absence and presence of EGF to measure total and non specific binding, respectively (Fig. 5). It was found that the SBR of EGF-NIR binding to COLO 205 is significantly higher than that of HT 29, and lower than that of A431, in accordance to EGFR 170 kDa protein isoform expression level (Fig. 5-insert). Although the SBR of total binding of CRC clones was between 3–6 (about 3 fold lower than A431), the non specific values of SBR between 1–2 allowed calculations of specific binding (NIR intensity) values of 3–4, which are in the sensitivity range of NIR detection systems [31], [32].

### BOI of Tumors Positive for EGFR in Orthotopic Mice Model

To confirm the suitability of EGF-NIR for in vivo BOI, HT-29 CRC orthotopic tumors in nude mice were generated (Fig. 6A). Western blotting analysis of dissected tumors confirmed the expression of EGFR in the tumors (Fig. 6A). Figs. 6B presents typical NIR fluorescence images of mice bearing EGFR positive tumors 4, 24 and 48 hours after i.v. injection of 1 nmol EGF-NIR, an optimal dose which affords the highest SBR, clearance and imaging results [28]. The control groups were i.v. injected first with 1 µg/ml cetuximab to block EGFR in orthotopic tumor and tissues [28] and after 5 hours injected with 1 nmol of EGF-NIR. In the first four hours the animal injected with the imaging agent show a very strong whole body fluorescence signal. After 24 hours, about 80% of the fluorescence signal cleared, and after 48 hours the signal intensity fell back to background level, in contrast to EGF-NIR labeling in tissues expressing EGFR: tumor, bladder and liver (Fig. 6B right image, taken at high resolution). Quantitative 2D surface measurements of the SBRs 4, 24 and 48 hours after injection, at each pixel (mm^2^), on ROI taken from the tumor and liver region, compared to an identical area on the flank (adjacent muscle) region are presented in Fig. 6C. It is evident that already after 24 hours EGF-NIR is specifically (competitive with cetuximab) and significantly accumulating in the tumor, providing an SBR value around 4.2 ± 0.6. This value is further increased at 48 hours. In the liver, a tissue highly abundant in EGFR [16], a significant accumulation or slowly clearance was observed after 48 hours and EGF-NIR binding was fully competitive with i.v. injected cetuximab. We assume that the fast accumulation at 24 hours of EGF-NIR reflects the very high level of EGFR in the tumors responsible for its uptake. Macroscopic 3D NIR estimations of harvested organs and urine were performed 48 hours after injection (Fig. 6D). The urine was strongly fluorescent, since EGF is known to be excreted unmodified into the urine [33], providing the highest SBR NIR fluorescence compared to that of the muscle. The liver/muscle SBR NIR fluorescence strongly exceeded compared to tumor (Fig. 6D) since the NIR intensity of the isolated tissue is higher than its value upon scanning the whole animal. Tumor/ muscle SBR NIR fluorescence was 11.3 ± 1.7 while the SBR 2D measurements of the whole animals was 6.6 ± 1.1. NIR imaging of isolated organs, in close proximity with the laser instrument, is more sensitive than the imaging of the organs in the animals, due to the lack of absorbance and scattering of the NIR fluorescence by the animal tissues [28].

### Specificity and Heterogeneity of CRC Tissue Upon ex vivo NIR Imaging

Figs 7 and 8 present *ex vivo* NIR imaging of human CRC tissues performed with EGF-NIR and processed by high resolution imaging to emphasize the distribution of EGFR. The binding experiments were conducted with fresh slices from CRC tissues identified by western blotting as either EGFR positive or EGFR negative (Fig. 7-insert). We also assessed slices from adjacent, colonic tissue close to the tumor, identified as EGFR negative in the same way previously performed for detection of CRC associated transcript-1 biomarker in malignant and pre-malignant CRC human tissues [24]. The binding was performed for 45 min to allow optimal diffusion of EGF-NIR into the slice. Significant total and specific binding of EGFNIR imaging agent was detected only in the CRC tissues positive for EGFR (Fig. 7). The ratios between specific binding of EGF-NIR to “CRC tissue EGFR+/adjacent colon EGFR-” or “CRC tissue EGFR+/CRC tissue EGFR-” were 46 and 16 (p < 0.05), respectively. These high values are easily detected by NIR instruments scanners. Fig. 8 presents 5 slices randomly chosen from the different tissues and their NIR imaging was processed by high resolution analyses. Spectral intensity maps of the slices indicate distinct focal areas of high level of expression of EGFR in CRC tissues EGFR+, with very high specific NIR labeling, few high spots of EGFR in adjacent colon tissue EGFR-with practically no specific NIR labeling, and very low level of EGFR in CRC tissue EGFR-with extremely low specific NIR labeling. These findings indicate a direct relationship between EGFR expression and EGF-NIR BOI and further indicate heterogeneity of CRC tissues in EGFR expression analyzed by spectral imaging software.

## Discussion

A new platform of NIR reagents based on IRDye 800CW was developed and used for preparation of IRDye 800CW conjugated EGF [27], [28], [34] which was accepted by NIH database as a molecular imaging and contrast agent for optical visualization of prostate carcinomas *in vitro* and in mice. We took advantage of this progress to prepare an EGF-NIR bio-imaging agent according to this procedure, and evaluated its pharmacological properties for recognition of EGFR in novel CRC models *in vitro*, resembling tumor heterogeneity, orthotopic CRC tumors in mice and CRC tissue slices *ex vivo*, thus translating results from CRC cells to human tissue specimens. EGF-NIR was homogeneous and pharmacologically active, as evident from binding experiments using CRC models. It specifically and selectively binds EGFR but not NRG 1 and its binding at 37°C was significantly higher than at 4°C, indicating that it is internalized at 37°C as expected from native growth factor. The binding of the probe measured by SBR directly reflected the level of EGFR, as evident from binding experiments with cells expressing lower EGFR levels, due to siRNA knockdown of EGFR and experiments with CRC cultures expressing different levels of EGFR. The EGF-NIR, rapidly saturated the EGFR in a focal CRC set-up, and was able to detect with high sensitivity 15–30% of EGFR expressing cells in a heterogeneous mixture of carcinoma/enterocyte cells *in vitro*. Consistent with the *in vitro* findings, specific targeting of EGF-NIR to EGFR was demonstrated by analyzing the NIR images of mice bearing EGFR positive CRC orthotopic tumors. Selective accumulation of EGF-NIR as evident from *in vivo* competition with cetuximab, was clearly seen after 24 and 48 hours in the tumor and EGFR positive organs such as the liver. The measurements of EGF-NIR in the tumors normalized to skeletal muscle and compared to the liver, indicated a faster accumulation of the agent in the tumor, compared to the liver, supporting the possibility of a specific EGFR- mediated process. These findings are reminiscent of accumulation studies using EGF- conjugated to Cy5.5 fluorophore in mice with breast cancer xenografts [16] and experiments of confocal endomicroscopy targeting EGFR with fluorescently-labeled antibodies [35]. We found relatively high SBRs for EGF-NIR binding to the whole animal and isolated tumors, supporting the notion that EGF-NIR is a suitable imaging agent for CRC tumor visualization using NIR endoscopy. This conclusion is also in line with studies in which dual labeling of a peptide with IRDye800CW and In^111^, generated satisfactory SBRs of 1.6 for NIR and 1.7 for nuclear imaging providing high quality images of the tumors in mice with human melanoma xenografts [36]. As documented by Goetz et al. [35], which measured a factor of 10 fold signal distinction of neoplastic from non-neoplastic CRC tissue using confocal laser endomicroscopy, we would like to stress that SBR value of 10 for isolated organs, scanned with the Odyssey Infrared Imager, represent a much higher value than the sensitivity threshold required by a NIR endoscope. SBR is an important parameter influencing the sensitivity of detection. Since EGFR is overexpressed in CRC tissue, it enables upon binding EGF-NIR, a relatively high SBR, facilitating detection with NIR scanners.

Following satisfactory results obtained in mice, we further characterized the applicability of EGF-NIR for *ex vivo* NIR imaging of human CRC tissues. Mouse and human EGF show more than 70% homology [37], therefore the EGF-NIR probe efficiently cross reacts with both mouse and human EGFR, enabling experiments with both mouse and human CRC tissues. We proved its specific binding to CRC tissue-EGFR positive, but not CRC tissue-EGFR negative and adjacent colon tissue-EGFR negative (Fig. 7 and 8), as previously demonstrated using FITC-labeled anti-EGFR antibody imaging of CRC with confocal endomicroscopy [35]. The ratios between specific binding of EGF-NIR to “CRC tissue-EGFR positive/CRC tissue-EGFR negative” or “CRC tissue-EGFR positive/adjacent colon tissue-EGFR negative” were 46 and 16 (p < 0.05), respectively. These high values prove that it is possible to generate a strong NIR fluorescence signal to detect fresh, unfixed CRC tissue-EGFR positive, using NIR scanners, as previously documented with an anti CEA antibody labeled with indocyanin green (ICG) NIR-Dye [31]. The heterogeneity of EGFR distribution among different slices and in the same slice of CRC tissue as observed in the present study may explain previous reports on the lack of correlation between clinical response to EGFR protein expression on immunohistochemical analyses of patients with refractory metastatic CRC [38], [39]. Since in the clinic, during the tissue processing, CRC EGFR may lose affinity upon handling and fixation, and antibodies used for diagnosis recognize epitopes different then EGF binding domain, we would like to propose that EGFNIR imaging of CRC fresh tissue slices may complement the above method [40]. This possibility needs to be further addressed using a larger cohort of human CRC tissues, in parallel with immunohistochemical analyses.

On one hand, EGF-NIR is significantly smaller then antibodies, it has higher tissue permeability, and its clearance as unbound agent can be achieved with simple washing steps, compared to antibodies labeled with NIR probes. Also its use can be more accurate in measuring EGFR expression than cetuximab in CRC tissue with variable affinity of EGFR for cetuximab [41]. On another hand, the risk in using such an agent is its ability to activate EGFR mediated downstream signaling such as RAS-Erk required for tumor proliferation. This problem can be solved by future designing of biomimetic peptides with reduced proliferative activity compared with native EGF or using EGFR targeting nanoparticles. Present findings provide EGF-NIR as an enabling platform technology for possible implementation of the binding protocol to visualize *ex vivo* EGFR in human CRC tissue, complementary to the gold standard technique of RT-PCR for EGFR mRNA quantification [6], in situ hybridization [8] and EGFR immunohistochemistry [7].

Fluorescence optical imaging has become a valuable tool as an adjunct to fiber-optic endoscopy due to its low cost and its ability to track multiple probes in a real time manner. Compared with the visible spectrum, the NIR dyes overcome the endogenous autofluorescence, maximize tissue penetration and are suitable for non-invasive whole body and organs imaging in small animals [17]. Over the last decade, NIR endoscopes were developed for high resolution imaging in small animals [42] and in identification of hyperplastic and adenomatous polyps in the colon [43]. The instruments currently available and/or under development, for clinical human imaging, such as Zeiss Pentero, with the microendoscope consisting of 20-gauge fiber optic catheter and dichroic beam splitters that simultaneously display visible light and 700 nm and 800 nm NIR fluorescent are used for intra-operative vascular flow and vascular surgery [44]. This instrument follow up the endogenous NIR autofluorescence of hemoglobin in the blood or of the FDA approved fluorescent ICG dye, either as a free molecule injected i.v. or conjugated to a relevant antibody such as anti-CEA and anti-mucin antibodies in local application [45]. Therefore, it is anticipated that present results, achieved with Odyssey Infrared Imager, will be reproduced with fiber-optic NIR endoscopes.

In conclusion, our study underscores the potential benefits of NIR optical imaging for BOI of EGFR in CRC tissues as evident from cell lines models *in vitro*, orthotopic tumors in mice at low dose (1 nmol/mouse) and *in situ* human tissues. The results suggest that BOI of EGFR using EGF-NIR probe may provide a sensitive, highly selective, non invasive tool for the detection and characterization of EGFR expressing CRC tissues without the need of radioactive imaging. This technology may complement immunohistochemical assessment of EGFR protein expression in CRC tissue which might contribute to future standardization methods measuring EGFR protein level, to improve the identification of patients who will benefit from anti-EGFR monoclonal antibody therapy.
